# Effects of Pre-Stretching on Creep Behavior, Mechanical Property and Microstructure in Creep Aging of Al-Cu-Li Alloy

**DOI:** 10.3390/ma12030333

**Published:** 2019-01-22

**Authors:** Jin Zhang, Zhen Jiang, Fushun Xu, Mingan Chen

**Affiliations:** 1 Light Alloy Research Institute, Central South University, Changsha 410083, China; zhangjin19861003@csu.edu.cn (J.Z.); jiangzhen2017@csu.edu.cn (Z.J.); machen@csu.edu.cn (M.C.); 2 Kunming Metallurgical Research Institute, Kunming 650031, China; 3 State Key Laboratory of High Performance and Complex Manufacturing, Central South University, Changsha 410083, China; 4 State Key Laboratory of Pressure Hydrometallurgical Technology of Associated Nonferrous Metal Resources, Kunming 650031, China

**Keywords:** Al-Cu-Li alloy, creep aging, pre-stretching, microstructure, mechanical property

## Abstract

The effects of pre-stretching on creep behavior, mechanical properties and microstructure during the creep aging process of Al-Cu-Li alloy were investigated. AA2195 was taken as the representative of Al-Cu-Li alloys. It is found that the total creep strain and strength property of creep aged AA2195 specimens can be improved through effective pre-stretching. Unlike with artificial aging, yield strength increased increasing by 47%. The TEM images show that the constitution of aging precipitates in the creep-aged specimens are obviously changed by pre-stretching. Precipitates in the 2% pre-stretched specimen are mainly composed of T_1_ phase, while a great amount of θ’ phase accompanied with a few T_1_ phase were found in the non-pre-stretched specimen. Moreover, pre-stretching introduces many dislocations which benefit the creep deformation, but the increasing dislocation density also accelerates the nucleation and growth of the precipitates as well. The premature T_1_ phase has a great blocking effect to the dislocation motion, creating a lower decrease rate but a longer duration in the early creep stage. Except for the initial dislocations, the dislocation motion in the creep aging process is also a favorable factor to precipitate the T_1_ phase.

## 1. Introduction

As one of the currently popular aeronautic and astronautic structural materials, Al-Cu-Li alloys have received worldwide attention and been developed rapidly in relevant production and application for their valuable combination of properties, such as low weight, high modulus and high specific strength [1,2,3]. As an excellent representative of Al-Cu-Li alloys, AA2195 has replaced the incumbent structural material (AA2219) for the cryogenic fuel tank on the space shuttle, as it provides an effective way of lowering the structural weight. In general, the T_1_ phase formed in aluminum {111} planes is considered as the primary strengthening phase in Al-Cu-Li alloys [4,5,6].

Plastic deformation prior to aging enhances the aging kinetics and increases the number density of fine strengthening precipitates through the introduction of dislocations acting as preferential heterogeneous matrix nucleation sites for the T_1_ phase [7]. Cassada et al. [8] found that the volume fraction of fine matrix T_1_ phase is enhanced by the effect of pre-deformation. Kim et al. [9] also found that the stretching treatment greatly accelerated the nucleation kinetics of the T_1_ phase at the expense of S’ phases. Gable et al. [10] conducted quantitative research and found that the size and quantity of the two Cu-containing strengthening precipitates (θ’ phase and T_1_ phase) of the Al-Cu-Li-X alloy will vary with the degree of change of pre-deformation in the same aging treatment. Matrix precipitation of this more abundant and finer strengthening phase directly correlates to increased strength and ductility of the material versus a non-stretched condition [11,12,13]. Dorin et al. [14] systematically studied the relationship between precipitate microstructure and resulting yield strength in the Al-Cu-Li alloy by combining the effect of pre-deformation, aging time and aging temperature, and these preceding findings were well considered in their microstructure-strength model.

Creep age forming (CAF) is an advanced metal forming method for manufacturing large integrally stiffened, lightweight structures of aluminum alloy. It synchronizes metal creep and age strengthening of aluminum alloy, thus greatly improving manufacturing efficiency. Examples of creep age forming application include the upper wing skins of Gulfstream IV/V, B-1B long-range combat aircraft, and Airbus A330/340/380 [15,16,17]. Furthermore, it has great potential in future large civilian/military aircraft manufacturing. During the creep aging process, the creep deformation and strengthening precipitates of aluminum alloy are constantly changing, and both interact with each other [18,19]. Compared with the artificial aging process without loaded stress, the evolution of microstructure and mechanical property appears more complicated in the creep aging process. This also means that, as a production efficiency advantage, some effective regulating methods for creep deformation and mechanical properties are necessary in the CAF process. According to previous research results, pre-deformation has great potential as a good regulating method for the creep aging of Al-Cu-Li alloys. Therefore, the effect of pre-stretching on the microstructure and mechanical property in creep aging of an Al-Cu-Li alloy was studied to provide a basis for application of CAF in the Al-Cu-Li alloy.

## 2. Materials and Methods

**Figure 1:**
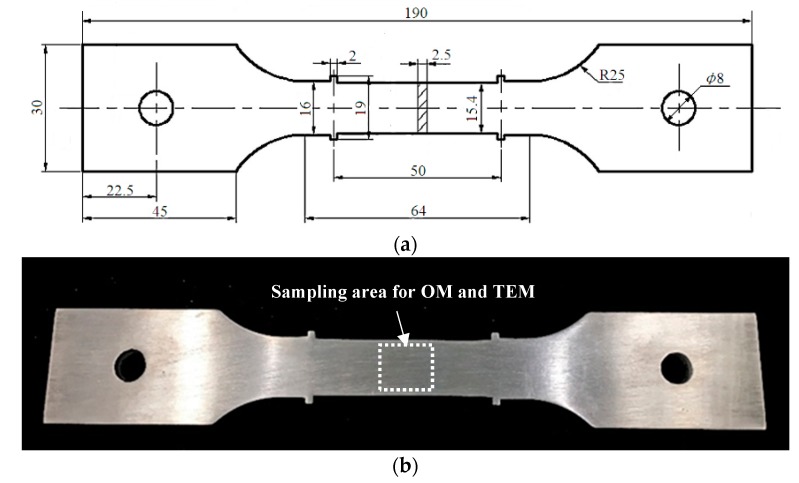
(**a**) Specimen geometry for creep aging tests (unit: mm); (**b**) sampling area of the microstructure specimens (OM and TEM).

The Al-Cu-Li alloy material used in this work was AA2195 hot rolled plate, and the chemical compositions are listed in Table 1. The content of each element is quantified in percentage of mass fraction (wt. %). Sheet specimens with a thickness of 2.5 mm were cut along the rolling direction of the hot rolled plate, and then they were machined into standard creep stretching specimens as shown in Figure 1a, with a gauge length of 50 mm.

The specimens were immediately cooled to room temperature through water quenching after being kept in a 510 °C solid-solution for 1 h. The temperature of the solid-solution was accurately controlled within ±2 °C. After quenching, some of the creep specimens rapidly underwent a 2% degree of pre-stretching at room temperature on the CSS-44100 electronic universal testing machine, and each pre-stretching process was carried out for 20 min. Meanwhile, the non-pre-stretched specimens were chosen for comparison. The metallographic and transmission electron microscope observation was conducted for some specimens at solid-solution state and pre-stretching state. Then pre-stretched and un-pre-stretched specimens were transferred to the RWS50 electronic creep relaxation testing machine equipped with a heating furnace device, where the uniaxial creep stretching test of constant stress and temperature was carried out. The thermocouple was placed in the middle of the specimen to ensure aging temperature. The temperature gradient along the gauge length of the specimen was controlled at 0.5 °C, and the required maximum allowable value was 3 °C. After the specimen reached the specified aging temperature and became stable, an extensometer was installed and the instrument was adjusted to apply a permanent load for recording displacement. The aging temperature was controlled at 170 °C for 24 h and the loading stress was 200 MPa. As a comparison, the parallel artificial aging specimens were also placed in the heating furnace device.

Tensile tests were carried out at room temperature using the CSS-44100 electronic universal testing machine at a constant crosshead speed of 2 mm/min. The measured value was equal to the average value of 6 specimens. The optical microscope (OM) specimens were treated with anodic coating after electrolytic polishing, and then examined under a DSX500 microscope. The specimens used for transmission electron microscope (TEM) observation were first mechanically thinned to the thickness of 80 μm and then twin-jet electropolished in a solution of 30% nitric acid and 70% methyl alcohol (in volume) at a voltage of 15 V and a temperature of −30 °C. The position of the microstructure specimen (OM and TEM) extraction in a creep-aged specimen is shown in Figure 1b. In addition, supplementary specimens were thinned to 200 nm thickness for dislocation density measurements. Finally, the microstructures of different specimens were observed using the Tecnai G220 transmission electron microscope operated at 300 kV.

## 3. Results

### 3.1. Creep Behavior

**Figure 2:**
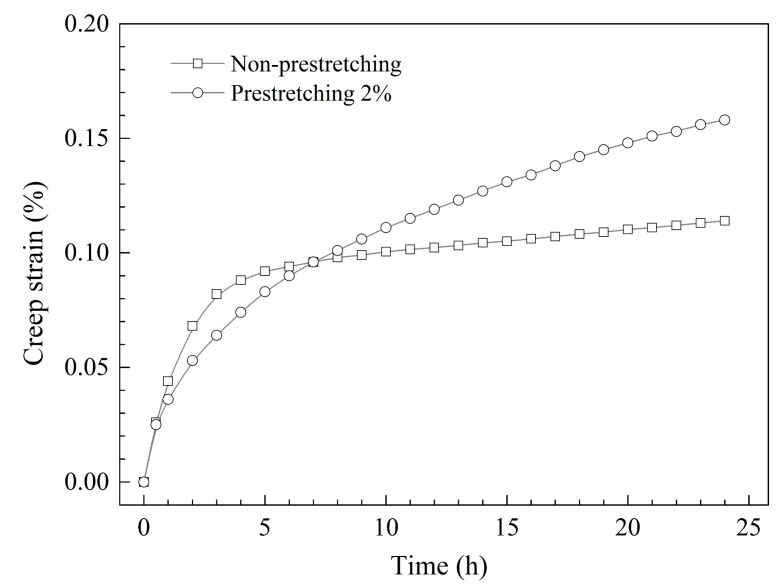
Creep strain curves of AA2195 specimens with different pre-stretched states under the same stress (200 MPa) and temperature (170 °C).

The creep strain curves of specimens in different pre-stretched states of AA2195 for 24 h under the same stress and temperature are shown in Figure 2. Overall, the creep strain curve of the pre-stretched specimen shows similar variation to the non-pre-stretched specimen in the test time, although the total creep strain of the pre-stretched specimen is clearly higher than the non-pre-stretched one. Creep rate is very fast in the initial stage, and then it decreases rapidly as the creep strain increases and gradually becomes stable. Finally, it keeps a stable slope and continues to increase. According to the character of the creep strain curves, it can be divided into three stages: initial rapid creep stage, intermediate slower creep stage, and steady creep stage.

In the initial rapid creep stage, there is no significant difference in the growth rate of specimens with pre-stretching or non-pre-stretching. In the intermediate slower creep stage, the creep strain of the pre-stretched specimen becomes lower than the un-pre-stretched one, firstly due to its earlier starting time at this stage. However, the pre-stretched specimen has a lower decrease rate and longer intermediate duration than the un-pre-stretched one, so that the lower creep strain soon catches up with the latter. It was noticed that, during the test time of 24 h, the creep strain of both the pre-stretched and non-pre-stretched specimens were mainly from the initial rapid creep and intermediate slower creep stages, where the creep strain reached 70% and 81% of the final totals respectively. In the steady creep stage, the creep rate maintained a lower constant value. In spite of this, the steady creep rate of the pre-stretched specimen was considerably higher than that of non-pre-stretched one, which was about 3 times as high as that of the latter. Therefore, pre-stretching can effectively improve the total creep strain by extending the duration of the intermediate slower creep stage and increasing the steady creep rate.

### 3.2. Mechanical Property

**Figure 3:**
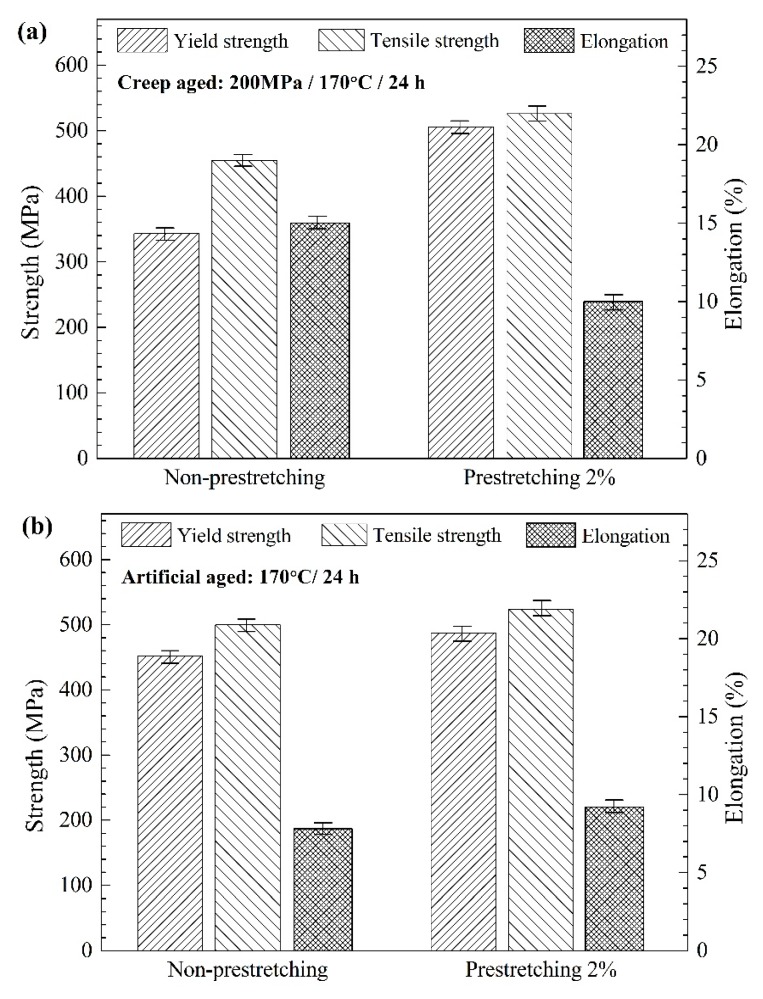
Comparison of mechanical properties of AA2195 specimens with different pre-stretching states after the process of: (**a**) creep aging and (**b**) artificial aging.

Figure 3 shows the comparison of mechanical properties of AA2195 specimens with different pre-stretched states after creep aging (Figure 3a), and also the reference specimens of the artificially aged states (Figure 3b). With the artificially aged states, both strength property and elongation of the pre-stretched specimen are higher than the non-pre-stretched. The promotion effect has been attributed to the enhanced aging kinetics of plastically deformed material and the changed competitive matrix precipitation, which promotes the increasing volume fraction of the primary strengthening phase (T_1_) [10].

With the creep-aged states, the strength property of the pre-stretched specimen is still higher than the non-pre-stretched. The difference is that the strength increase appears much larger, as the increased rate of yield strength reached 47%. This is because of the obviously lower yield strength of the non-pre-stretched specimen within the creep-aged state. The average value of 6 parallel samples is only 343 MPa, which is the lowest value of all the test conditions. Thus, it implies that the strength property of the non-pre-stretched AA2195 specimens was significantly decreased by the creep aging process. However, the 2% pre-stretched specimen with creep-aged state, by contrast, reached the strength level of 500 MPa in addition to a significant elongation under the present conditions. Therefore, the pre-stretching, which brings advantages to the aforementioned creep strain and also the room-temperature mechanical property, should be an indispensable process in the creep-age forming of the AA2195 alloy.

### 3.3. Microstructures

**Figure 4:**
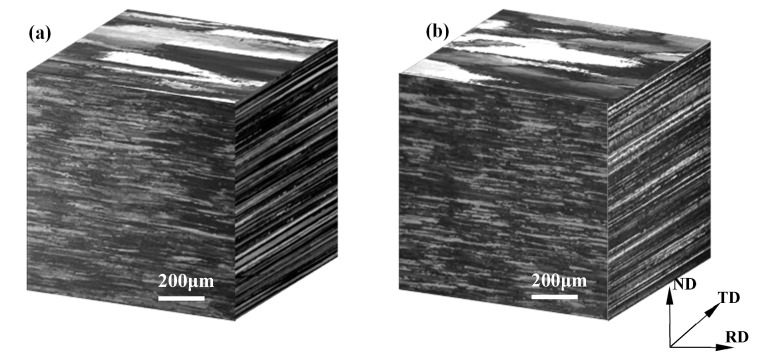
Optical micrographs of AA2195 specimens: (**a**) non-pre-stretching and (**b**) pre-stretching 2%.

**Figure 5:**
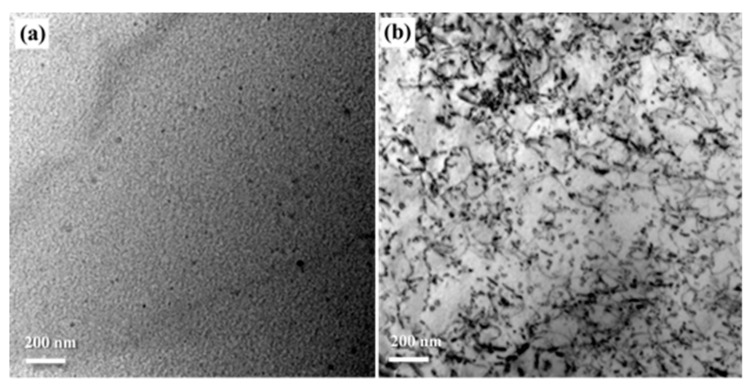
TEM images of dislocation morphology of AA2195 specimens: (**a**) non-pre-stretching and (**b**) pre-stretching 2%.

Figure 4 shows metallographic images of non-pre-stretched and 2% pre-stretched specimens after solid-solution and water-quenching. There is no obvious difference between them; both show similar grain morphology of the elongated shape in the direction of rolling, accompanied by fine equiaxed grains. The effect of the deformation degree of 2% did not appear in the grain morphology but increased the intracrystalline dislocation density. As shown in Figure 5a, there are only a few dislocations in the grains of the solid-solution state specimen without pre-stretching. However, in the 2% pre-stretched specimen, as shown in Figure 5b, numerous scattered bending dislocation lines can be observed.

**Figure 6:**
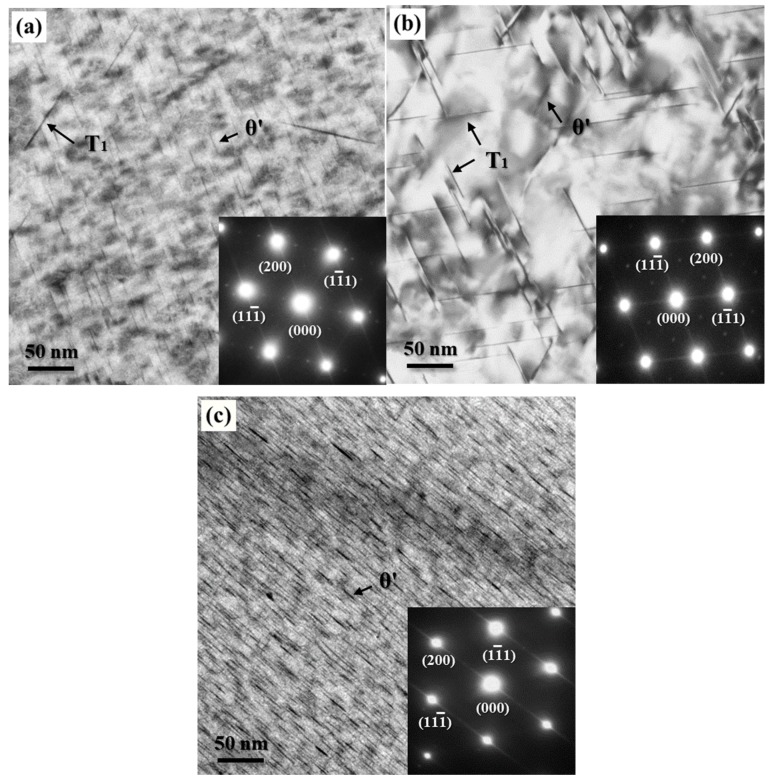
TEM images and SAED spots along [110]_Al_ zone axis of AA2195 specimens: (**a**) creep aged with non-pre-stretching; (**b**) creep aged with pre-stretching 2%; and (**c**) artificially aged with non-pre-stretching.

TEM images and SAED spots along (110) [110]_Al_ zone axis of the non-pre-stretched and 2% pre-stretched specimens after creep aging for 24 h are shown in Figure 6a,b respectively. T_1_ phase can be readily identified from the reflections at 1/3<220> and streaks along the <111> direction in the [110]_Al_ SADP, while streaks along the <200> direction in the [110]_Al_ diffraction patterns indicate the presence of fine θ’ precipitates. It is obvious that the constitution of precipitation differs greatly between the two kinds of specimens. Precipitates in the 2% pre-stretched specimen were mainly composed of T_1_ phase, while a great amount of θ’ phase as well as a few T_1_ phase were found in the non-pre-stretched specimen. This θ-based phase constitution appears to be more evident in the TEM images of the artificially aged specimen with non-pre-stretching, as shown in Figure 6c. Very dense θ’ precipitates distribute in the matrix with almost no T_1_ precipitates. Generally, the elongation increases at the expense of the tensile strength, as evidenced by the creep-aged specimens with different pre-stretching states (Figure 3a). However, it seems that the extreme phase transformation as shown in Figure 6c not only causes a decrease in tensile strength, but also abnormally low elongation compared to the pre-stretched specimen at the artificially aged state, as shown in Figure 3b. For the growth of the intracrystalline T_1_ precipitates were severely inhibited, the precipitates containing Li can be enriched at the sugrain boundaries. Additionally, the ductility of the specimen was markedly decreased for this reason [20].

Furthermore, comparing the two types of non-pre-stretched specimens (artificially aged and creep-aged), it can be deduced that the phase constitution of the creep-aged specimen with non-pre-stretching appears to have an obviously lower strengthening effect than others. For a small amount, T_1_ precipitates can also consume the Cu atoms in the matrix, which leads to a decrease in θ’ precipitates thereby widening phase spacing. Thus, the corresponding strengthening effect is less than the status of the pure but dense θ’ phase.

## 4. Discussion

### 4.1. Pre-Stretching and Creep Strain

**Figure 7:**
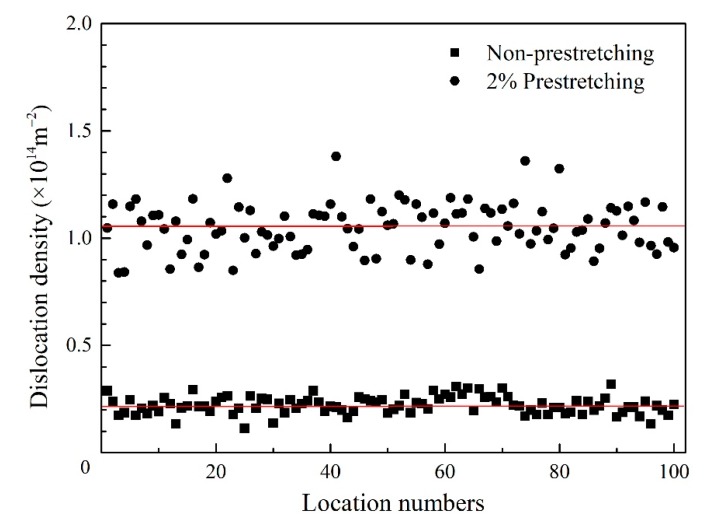
Measured dislocation density of the non-pre-stretched and 2% pre-stretched AA2195 specimens.

To investigate further, the dislocation density of the two kinds of specimens was also measured by the line intersection method [21] using a great many TEM-micrographs. A grid consisting of four horizontal and four vertical lines was superimposed on different areas of dislocation lines, and the dislocation density *ρ* can be obtained from the following calculation formula:

*ρ* = (Σ*n_v_*/Σ*L_v_* + Σ*n_h_*/Σ*L_h_*)/*t* (1)

where the number *n_v_* and *n_h_* are intersections of dislocations with horizontal and vertical grid lines respectively, Σ*L_v_* and Σ*L_h_* are the total lengths of the horizontal and vertical test lines from all micrographs taken for one material state respectively. In Figure 7 we present the results obtained from 100 different locations for both the non-pre-stretched and 2% pre-stretched specimens. The mean value of each state is marked by a horizontal line and it can be seen that the dislocation density *ρ* was increased by pre-stretching from 2.21 × 10^13^ m^−2^ to 1.05 × 10^14^ m^−2^.

According to the experimental results and analysis in Section 3.1, it is already known that the creep deformation mainly comes from the initial rapid creep stage and intermediate slower creep stage under the test conditions, as the creep develops mainly from the rapid slipping of dislocation under stress in the initial stage. Additionally, the creep strain of the pre-stretched specimen surpasses the un-pre-stretched one quickly with a longer duration during the second stage, because the increased mobile dislocations in the initial material matrix of the pre-stretched specimen can supply enough deformation in the early creep stage. Moreover, due to increased dislocation motion, involving dislocation multiplication and recovery, the creep rate of the pre-stretched specimen stabilizes at a higher level during the steady creep stage. Therefore, the increased creep strain of the pre-stretched specimens should be attributed to the abundant mobile dislocation introduced by pre-deformation.

### 4.2. Pre-Stretching and Precipitation

**Figure 8:**
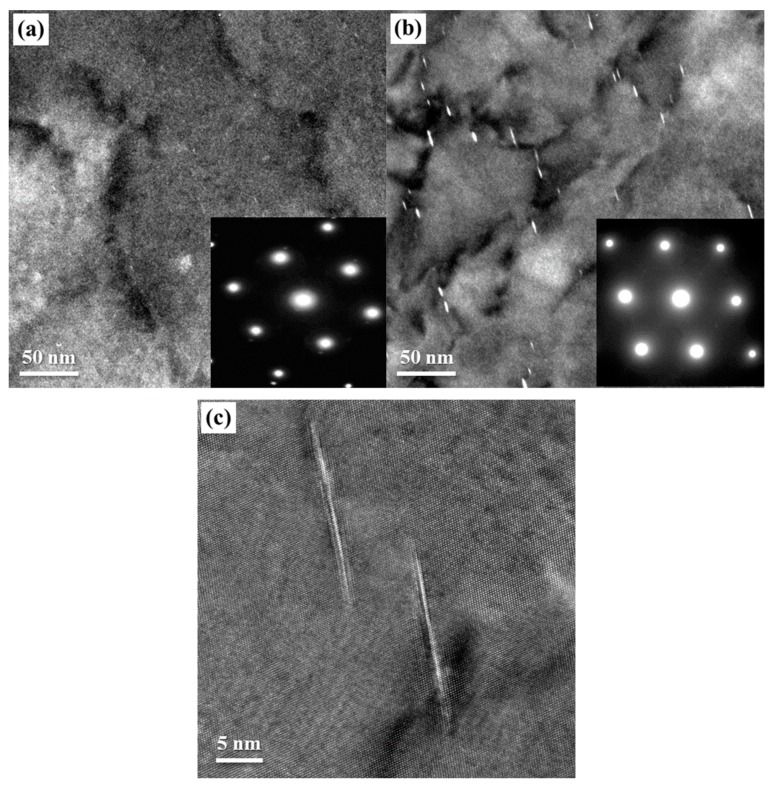
TEM images and SAED spots along [110]_Al_ zone axis of AA2195 specimens after creep aging for 2 h: (**a**) non-pre-stretching; (**b**) pre-stretching 2%; and (**c**) pre-stretching 2% (HRTEM).

The increase of the initial dislocation density, which is introduced by pre-stretching, can accelerate the nucleation and growth of the T_1_ precipitates during the aging process [9,10,11,12]. Figure 8a,b shows TEM images and SAED spots of the non-pre-stretched and 2% pre-stretched specimens after creep aging for 2 h, and Figure 8c shows the corresponding HRTEM image of the latter. In the specimen with non-pre-stretching, there are almost no visible precipitates in the matrix. However, in the specimen with 2% pre-stretching, visible T_1_ precipitates can be clearly seen on or around the matrix dislocation lines, and weak streaks along the direction of <111> are observed in the corresponding SAED. Combined with the creep curve in Figure 2, we can find that the premature precipitation of the T_1_ phase coincided with the decreasing creep rate during the initial stage. Thus, it can be deduced that the premature T_1_ precipitates should have an obvious blocking effect to the dislocation motion in the early creep stage, making a rather slow creep deformation velocity in the intermediate slower creep stage.

The atomic structure of the T_1_ phase and the T_1_ precursor phase have been the subject of extensive recent study [22,23,24], and T_1_ precursor precipitates were also found in the dislocation-free zone. These studies have expanded on the previous theory of T_1_ phase structure models based on the stacking of {111} type planes by dissociation of perfect dislocations into partials [6]. However, it is clear that the T_1_ phase nucleates grow preferentially at lattice defects (such as dislocations and sub-boundaries), as well as the corresponding nucleation and growth mechanism. Based on this viewpoint, the increased duration of the intermediate slower creep stage of the pre-stretched specimen in this paper can be reasonably explained. The nucleation of the T_1_ phase preferentially occurs in stacking faults on {111} planes, where the segregation of Cu and Li atoms forms “Suzuki atmosphere”, thus hindering dislocation motion in the nucleation stage. Clearly, the Suzuki interaction force will gradually disappear along with the end of nucleation, and the dislocation motion in the initial creep stage will quickly return to a high level. In addition, this hindering effect upon dislocation motion can only manifest clearly along with a mass of dislocations and a sufficient amount of nucleation due to the small Suzuki interaction force.

The experimental results also indicate that the precipitation constitution of the creep-aged specimens can be significantly changed by pre-stretching. A large amount of the θ’ phase accompanied with a few of the T_1_ phase were found in the non-pre-stretched specimen, but the precipitates in the 2% pre-stretched specimen were mainly composed of T_1_ phase. This phenomenon can also be interpreted combining the microstructures at the early creep aging stage, as shown in Figure 8. For the 2% pre-stretched specimens, the T_1_ precipitates took the lead in accomplishing nucleation at the early creep aging stage. This wins significant advantages for the T_1_ phase in the subsequent competitive environments of the aging precipitation process. Nevertheless, most of the precipitation in the non-pre-stretched specimen needs to evolve from the GP zones. This process seems to be more conducive to the precipitation of θ’ phase. Moreover, with the initial microstructure having few dislocations and dislocation motions, the Cu atoms precipitated basically in the form of the θ’ phase (Al_2_Cu) during the aging process, and the form of the T_1_ phase (Al_2_CuLi) was suppressed almost completely, as shown in Figure 6c. But in the case of few dislocations alongside a certain amount of dislocation motions in the initial stage, it was observed that the Cu atoms partially precipitated in the form of the T_1_ phase (Al_2_CuLi), as shown in Figure 6a. It can thus be deduced that the dislocation motion in the creep aging process is also a favorable factor for the formation of T_1_ precipitation, in addition to increasing the initial dislocation density.

## 5. Conclusions

Based on the above results and discussions, the following conclusions can be made:

(1) The total creep strain of the Al-Cu-Li alloy can be improved by effective pre-stretching. In the intermediate slower creep stage, the creep strain of the pre-stretched specimen becomes lower than the non-pre-stretched one, primarily due to the earlier starting time of this stage. The lower creep strain soon catches up with the latter, because of the lower decrease rate and longer intermediate duration.
(2) The strength property of the pre-stretched Al-Cu-Li alloy specimen is much higher than the non-pre-stretched one after the same creep aging treatment. In contrast to the artificial aging process, the strength increase appears much larger, as the yield strength rises by 47%. The strength property of the non-pre-stretched Al-Cu-Li alloy specimen was significantly decreased by the creep aging process, but the room-temperature mechanical property of the creep-aged specimens with 2% pre-stretching is very close to the artificially aged specimens, and even slightly higher than the latter.
(3) Pre-stretching introduces lots of mobile dislocations which benefit the increase of the creep deformation, but the increasing dislocation density will accelerate the nucleation and growth of the precipitates as well. Premature T_1_ precipitation has a great blocking effect on the dislocation motion in the early creep stage, resulting in a markedly lower decrease rate but longer duration in the intermediate slower creep stage.
(4) The constitution of aging precipitates in the creep-aged specimens can be significantly changed by different pre-stretching states. The precipitates of the pre-stretched specimen are mainly composed of the T_1_ phase, while the θ’ phase consists of the main precipitates in the non-pre-stretched specimen. A small amount of T_1_ precipitates in the non-pre-stretched specimen leads to the decreasing amount and widening phase spacing of θ’ precipitates by consuming Cu atoms in the matrix, so that the strengthening effect appears lower than in the others. Except for the initial dislocations, the dislocation motion in the creep aging process is also a favorable factor to precipitate the T_1_ phase.

## Figures and Tables

**Table 1:** Chemical composition of 2195 aluminum alloy (wt. %).

Si	Fe	Cu	Mn	Mg	Zr	Ag	Li	Other	Al
0.03	0.04	4.1	0.04	0.28	0.13	0.26	0.9	<0.1	Bal
